# Secretion Systems and Secreted Proteins in Gram-Negative Entomopathogenic Bacteria: Their Roles in Insect Virulence and Beyond

**DOI:** 10.3390/insects9020068

**Published:** 2018-06-19

**Authors:** Rebecca McQuade, S. Patricia Stock

**Affiliations:** 1 Center for Insect Science, University of Arizona, 1007 E. Lowell Street, Tucson, AZ 85721, USA; rmcquade@email.arizona.edu; 2 Department of Entomology and School of Animal and Comparative Biomedical Sciences, University of Arizona, 1140 E. South Campus Dr., Tucson, AZ 85721, USA

**Keywords:** secretion systems, Gram-negative bacteria, host interactions, entomopathogens

## Abstract

Many Gram-negative bacteria have evolved insect pathogenic lifestyles. In all cases, the ability to cause disease in insects involves specific bacterial proteins exported either to the surface, the extracellular environment, or the cytoplasm of the host cell. They also have several distinct mechanisms for secreting such proteins. In this review, we summarize the major protein secretion systems and discuss examples of secreted proteins that contribute to the virulence of a variety of Gram-negative entomopathogenic bacteria, including *Photorhabdus*, *Xenorhabdus*, *Serratia*, *Yersinia*, and *Pseudomonas* species. We also briefly summarize two classes of exported protein complexes, the PVC-like elements, and the Tc toxin complexes that were first described in entomopathogenic bacteria.

## 1. Introduction

Entomopathogenic bacteria have developed numerous strategies to interact with, and kill, insects. Many of these involve specialized secretion systems, which transport various proteins from the bacterial cytosol to the bacterial surface, into the host environment, or directly into host cells, where they play diverse roles in promoting bacterial virulence. These can include evasion of host immune mechanisms, intoxication of host cells, and nutrient acquisition within the host [1,2]. Highlighting their importance in pathogenesis, some secretion systems are expressed in response to recognition of host receptors or other signals from the host environment [3]. Protein secretion in Gram-negative bacteria is complicated by the double-membrane architecture of their cell envelope, which has led to the development of elaborate secretion mechanisms. Genomic analysis has yielded a wealth of information about potential secretion mechanisms in entomopathogenic bacteria and other microbes, providing an excellent starting point for understanding this crucial aspect of their biology [4,5,6,7,8].

In Gram-negative bacteria, secretion systems can deliver proteins from the bacterial cytoplasm into the periplasm (the Sec and Tat systems), into the extracellular space (T1SS, T2SS, autotransporters, and two partner systems), or directly into target cells (T3SS, T4SS, and T6SS). Secretion systems differ in the kinds of protein substrates they transport, particularly whether the proteins are folded, and whether they have a signal peptide [2,3,9,10]. In general, the structural elements of the secretion systems discussed in this review have been identified, and some are clearly related to structures with other functions, such as the phage-like T6SS [11,12], and the T3SS injectisome, which is descended from the flagellar assembly complex [13,14].

In this review, we give a brief introduction to some of the major Gram-negative entomopathogenic bacteria, describe their identified secretion systems (summarized in Table 1), and provide examples of their secreted proteins (Table 2), focusing on those with experimentally verified roles in pathogenesis. We will not discuss T4SSs (associated with conjugation machinery) or the chaperone-usher pathway (mediating pilus assembly). We will also not discuss proteins thought to function primarily as adhesins, though these certainly qualify as secreted proteins and frequently contribute to virulence. Some genes thought to be important for insect pathogenesis appear to have been horizontally transferred among entomopathogens [15], and some of these encode secreted proteins. Where possible, we will compare homologs of secreted proteins from different entomopathogens to highlight the similarities and differences in their functions.

## 2. Gram-Negative Entomopathogens

Several excellent reviews provide a comprehensive account of entomopathogenic bacteria [16,17,18]. Here we will briefly sketch the lifestyles of some important species before exploring the contributions of secreted proteins to their virulence.

Two closely-related genera, *Photorhabdus* and *Xenorhabdus* live as obligate symbionts with Heterorhabditid and Steinernematid nematodes, respectively [7,19]. The nematodes vector the bacterial symbionts into a broad spectrum of soil-dwelling insect hosts, especially those in Lepidoptera and Coleoptera [20,21,22]. The bacteria, rather than the nematodes, have been generally recognized as contributing most to insect virulence [23,24,25,26,27,28]. Culture supernatants from *Photorhabdus* and *Xenorhabdus* contain a variety of toxins, exoenzymes, and antibacterials [29,30,31,32], some with demonstrated roles in insect killing, nutrient acquisition, immune evasion, and protection of the insect cadaver from competitors. In some cases, these proteins have been associated with particular secretion systems. Among *Photorhabdus* and *Xenorhabdus*, a single species, *Photorhabdus asymbiotica*, is known as an opportunistic pathogen of humans [6,33]. Its altered host range is thought to be due in part to differences in the proteins it secretes compared to other *Photorhabdus* spp. [34,35].

Several free-living bacteria from several genera are also potent entomopathogens. For example, insect-pathogenic *Serratia* spp. are capable of causing disease in a diverse range of insect orders including Coleoptera, Hymenoptera, Lepidoptera, and Diptera. In particular, *Serratia entomophila* and *S. proteomaculans* are responsible for amber disease in larvae of the grass grub *Costelytra zealandica* [36].

*Yersinia entomophaga* is a potent pathogen of Coleoptera, especially those in the scarab family [37], and its genome includes several likely insecticidal toxins. Though not an insect pathogen, *Yersinia pestis* does colonize fleas and its genome encodes potential insecticidal toxins as well [38,39]. Finally, *Pseudomonas entomophila* is pathogenic to a range of insects, especially *Drosophila melanogaster* [40], and this bacterium also encodes several proteins with likely insecticidal activity [5].

## 3. Identifying Secreted Proteins

The availability of genome sequences for representative strains of these entomopathogenic bacteria has facilitated bioinformatic analysis of their likely secretion systems. Predicting structural components of secretion systems is relatively straightforward, but can be complicated by their similarity to other structures, like phage (T6SS) and flagella (T3SS), and different algorithms can produce discrepant results [41,42]. For instance, the *Photorhabdus luminescens* TT01 genome has been reported to encode four or six T1SSs and six or eight two-partner systems, depending on the bioinformatics pipeline used [43,44]. The reliability of predictions for secreted proteins varies widely: proteins moved through the Sec, Tat, and T1SS usually have predictable, conserved signal sequences [45,46,47], while those secreted by T2SS, T3SS, and T6SS do not, making these more challenging to identify bioinformatically [48]. Inferences based on homology to proteins from other organisms should be considered starting places requiring experimental verification.

Several different kinds of evidence can suggest that a given protein is secreted through a particular secretion system: (a) the presence of an appropriate signal sequence (b) similarity to known secreted proteins (c) localization at the bacterial surface, in culture supernatants or in host cells (d) loss of localization in mutants lacking the suspected secretion system (e) secretion of reporter fusions; and (f) structural analyses [49]. Most of the examples in this review are supported by at least two of these; naturally, the strongest arguments combine the most lines of evidence.

## 4. Secretion Systems of Entomopathogenic Bacteria

### 4.1. Sec and Tat Secretion Systems: From the Cytoplasm into the Periplasm

Most Gram-negative bacteria, including insect pathogens, encode the Sec translocase and Twin-arginine translocation (Tat) pathways to move proteins from the cytoplasm across the inner membrane into the periplasm (the compartment between the Gram-negative inner and outer membranes). The Sec pathway is common to Gram-negative bacteria, Gram-positive bacteria, and eukaryotes. Proteins secreted by the Sec system are generally unfolded, have an easily identifiable N-terminal signal peptide, and are brought to the Sec machine by the molecular chaperone SecB [3,10].

The Tat secretion system moves folded proteins across the cytoplasmic membrane in both Gram-positive and Gram-negative bacteria. Its substrates have the characteristic sequence motif of two sequential arginine residues in their N-terminal signal peptide sequence. In Gram-negatives, some Sec- and Tat-secreted proteins remain in the periplasm. For instance, the antibacterial xenocins of *Xenorhabdus* spp. are coexpressed with periplasmic immunity proteins that protect *Xenorhabdus* from the lethal effects of their own xenocins [50]. In clinical isolates of *P. asymbiotica,* the amoxicillin resistance that can complicate treatment [51] is likely conferred by a variant β-lactamase moved into the periplasm by the Tat system [52].

### 4.2. Sec-Dependent Secretion Systems: From the Periplasm out of the Cell

Gram-negative bacteria have several strategies for transporting proteins from the periplasm across the outer membrane, after which they may be retained on the bacterial surface or released into the environment. These so-called “Sec-dependent” mechanisms include the T2SS, autotransporters, and two-partner secretion systems.

### 4.3. Type 2 Secretion System

In type 2 secretion, proteins are first translocated across the inner membrane by the Sec or Tat pathway, and then become targets of the T2SS in the periplasm. The T2SS machinery is normally comprised of 12–16 proteins, with components anchored in both membranes, and secretes folded proteins [55]. A consensus secretion signal for T2-secreted proteins has not been identified, and it has been proposed that the motif needed for secretion appears only in the folded protein [55]. In some strains of *P. luminescens*, the large insecticidal TcdA1/B1 toxin complexes (described below) appear to remain anchored at the bacterial cell surface, while, in others, they are released into the extracellular environment, leading to differences in oral toxicity of supernatants for insect larvae [61,62]. Two proteins described as T2-secreted proteins, as well as a lipase called Pdl, seem to be involved in processing Tc complexes at the bacterial cell surface [62].

### 4.4. Type 5 Secretion: Autotransporters and Two-Partner Secretion Systems

Type 5 secretion systems encompass a large family of proteins that facilitate their own transport from the periplasm out of the bacterial cell. These include classical autotransporters (T5aSS) and two-partner secretion systems (TPSs, also called T5bSSs), as well as several other structurally distinct variations [57,58]. Autotransporters are multi-domain proteins, usually over 100 kDa in size, encompassing an N-terminal Sec signal sequence, allowing them to move through Sec into the periplasm, and a C-terminal β-barrel domain, which forms a pore in the outer membrane, facilitating translocation of the passenger domain to the extracellular environment. Some autotransporters also include a protease domain to release the passenger from the bacterial surface into the environment. The passengers are structurally and functionally quite diverse, but most share a similar β-helical structure thought to facilitate their translocation. Many are virulence factors [57,58]. The *X. nematophila* lecithinase EstA includes conserved domains of an autotransporter, is expressed in vitro, and provides lecithinase activity. It is not required for virulence against tobacco hornworms (*Manduca sexta*), or for colonization of *Steinernema* nematodes, but it is required for growth when lipids are the sole carbon source [63], suggesting a role in later, nutrient-limited life stages. *Ps. entomophila* encodes a predicted autotransporter with a passenger domain similar to a *Yersinia pestis* hemolysin [5]. Its function is currently unknown.

TPSs are structurally and functionally similar to autotransporters, but involve two separate polypeptides, TpsA (the secreted protein) and TspB (the pore-forming translocator protein). Both proteins are moved into the periplasm by Sec, then TpsB forms a β-barrel structure in the outer membrane, allowing for TpsA to exit the cell. The TpsA partner generally has a recognizable N-terminal TPS domain that targets it for secretion through TpsB [57,60]. Kim et al. identified three groups of TPS homologues that are widespread in entomopathogens, including PhlA-type hemolysins, contact-dependent inhibition systems, and a third group with an as yet unknown function [77].

The hemolysin PhlA, along with its secretion partner PhlB, is one of several TPS pairs encoded by the *Photorhabdus luminescens* type strain TT01. PhlA is not required for *P. luminescens* virulence, but GFP-reporter assays indicate that it is expressed in insects during infection [64], possibly contributing to bioconversion of the cadaver. In *X. nematophila*, the homolog XhlA does contribute to virulence in the *M. sexta* model (likely through lysis of insect hemocytes) [65], and is transcriptionally regulated by the Lrp regulator and iron limitation. This is consistent with a specific role in the insect-pathogenic phase of the *Xenorhabdus* lifecycle. The contact-dependent inhibition TPS from *Xenorhabdus* spp., CdiAB, appears to follow the classic pattern for contact-depend inhibition [78], with the C-terminal portion of CdiA displaying antibacterial activity that is eliminated in the presence of the cognate immunity protein CdiI. These data suggest a role for CdiAB in interbacterial competition, likely in the context of protecting the insect cadaver from scavengers. However, the CdiAB locus appears to be pseudogenized in several *Xenorhabdus* and *Photorhabdus* genomes, consistent with these proteins providing an advantage only in certain niches [66].

### 4.5. Single-Step Secretion: From the Cytoplasm out of the Cell

Gram-negative bacteria also use several specialized mechanisms to move proteins directly from the cytoplasm into the extracellular space or directly into a target cell, bypassing the periplasm. These include the T1SS, T3SS, and T6SS, all of which are represented in entomopathogens’ genomes.

### 4.6. Type 1 Secretion System: From the Cytoplasm to the Extracellular Space

T1SSs are essentially ABC transporters complexed with two other components to move proteins of various sizes across both membranes of the Gram-negative cell envelope. The ABC (ATP-binding cassette) protein in the inner membrane provides the energy needed for secretion. A membrane fusion protein (MFP) couples this to an outer membrane channel protein (similar to TolC) allowing the export of substrates [47,53,79]. T1–secreted proteins vary greatly in size, but are generally unfolded and include a C-terminal signal peptide characterized by glycine-rich repeats [47]. This arrangement works well for large proteins, as it ensures that only completely translated substrates are secreted [47]. Many virulence proteins are exported by T1SSs, particularly proteins belonging to the functionally diverse RTX (repeats-in-toxin) protein family [53,54].

The *P. luminescens* genome encodes multiple putative T1SSs [4,44] and two of its best-characterized virulence factors, the “makes caterpillars floppy” toxin Mcf1 and the metalloprotease PrtA, are T1-secreted RTX-family proteins. Mcf1 is pro-apoptotic and strongly insecticidal [44,67,68]. Heterologous expression of Mcf allows injected *E. coli* (normally cleared by the larval immune system) to avoid encapsulation and cause the loss of turgor characteristic of *Photorhabdus* infection in *M. sexta* caterpillars [67]. Mcf promotes apoptosis in both insect hemocytes [67] and mammalian cells [68], suggesting the mechanism of its toxicity. Several *Xenorhabdus* species also encode a homolog of McF, which is likely moved through a T1SS [77].

The *Photorhabdus* metalloprotease PrtA is encoded in an operon with its presumptive T1SS, called PrtBCD, and a protease inhibitor [69]. An early report using zymography showed active PrtA in *Photorhabdus*-infected insect cadavers late in infection, suggesting a role in bioconversion [69]. PrtA is unable to cleave many structural proteins [69], but can cleave immune proteins from insect hemolymph, suggesting a role in immune evasion [70] rather than nutrient acquisition. A PrtA homolog in *X. nematophila* (also encoded alongside a T1SS) is expressed early in growth and protects *X. nematophila* from the antibacterial activity of insect hemolymph, suggesting a role in immune evasion in this bacterium [71]. The *Ps. entomophila* homolog, called AprA, also appears to be involved in immune evasion; an *aprA* mutant is rapidly cleared from wild-type *Drosophila melanogaster*, but not immune-deficient *Relish* flies, and purified AprA protease can cleave insect antimicrobial peptides [72]. The *aprA* mutant is also defective in killing flies [72,73]. AprA and the PrtA proteins belong to the larger T1SS-dependent RTX family [80]. RNA-Seq analysis indicates that the *P. asymbiotica prtA* and *mcf* homologues are increased in expression at 37 °C compared to 28 °C, suggesting that these factors participate in human infection [81].

### 4.7. Injectisome Type 3 Secretion System: From the Bacterial Cytoplasm into a Target Cell

One mechanism of protein secretion especially associated with pathogens is the T3SS injectisome. T3SSs include two classes: flagellar T3SSs responsible for assembling flagella, and non-flagellar or injectisome T3SSs, which deliver proteins from the bacterial cytoplasm directly into the cytoplasm of eukaryotic target cells. Both structures span the bacterial inner and outer membranes, and the two systems share a common core of nine structural proteins. Injectisome T3SSs include additional proteins for the translocation pore that inserts into the target cell membrane. Injectisome T3-secreted proteins are called “effectors,” and generally associate with chaperone proteins in the bacterial cytoplasm, which deliver them to the T3SS, where they are unfolded and secreted into target cells [13,14]. Known T3-secreted effectors usually have N-terminal secretion signals within their first 40 amino acids [13] (mapped by assessing the secretion of recombinant effectors with mutations in the N terminus, or of reporters fused to N-terminal fragments of different lengths), but there is little consensus among these signals, and they remain frustratingly difficult to predict bioinformatically. 

Genomic analyses have revealed extensive horizontal gene transfer of injectisome T3SSs among Gram-negative bacteria, including entomopathogens [56], and the carriage of T3SS genes varies widely among them. *Ps. entomophila* is the only sequenced *Pseudomonas* species to date lacking a T3SS injectisome [5]. Likewise, multiple genomic analyses report the absence of T3SSs in *Xenorhabdus* spp. [7,61,82,83].

All examined *Photorhabdus* strains encode at least one T3SS, with conserved gene content and synteny in the fourth Pathogenicity Island (PAI IV) of the genome [15]. The structural elements of this secretion system are similar to T3SSs of *Yersinia* spp. and *Pseudomonas aeruginosa* [15,34]. This T3SS inhibits phagocytosis of *P. luminescens* by hemocytes, decreasing the nodulation response and allowing the bacteria to persist in the insect hemocoel [74]. The putative T3SS effector LopT has demonstrated cytotoxic activity in vitro, but appears not to be essential for virulence [74]. Its similarity to *Yersinia pestis* YopT suggests it may play a role in evading phagocytosis. Another putative T3-secreted protein from *Photorhabdus* is Cif, a cycle-inhibiting factor similar to demonstrated T3SS effectors from the human pathogens enteropathogenic and enterohemorrhagic *E. coli*. *Photorhabdus* Cif causes cell cycle arrest and actin rearrangement in cultured cells, suggesting it is a toxin with activity similar to the pathogenic *E. coli* proteins [75].

Interestingly, the human pathogen *P. asymbiotica* encodes an additional T3SS in a separate genomic island not found in other *Photorhabdus* species, which resembles a locus from *Vibrio parahaemolyticus* [6]. *P. asymbiotica* lacks a LopT homolog, but has a likely T3SS effector similar to the strongly cytotoxic ExoU phospholipase of *P. aeruginosa*, and another called SopB similar to a *Salmonella* T3SS effector involved in host cell invasion [35]. These differences in T3SS gene carriage likely contribute to *P. asymbiotica*’s ability to infect humans.

A somewhat controversial example involving T3 secretion comes from *Yersinia pestis. Y. pestis* produces a Tc toxin complex comprising the YitABC proteins and likely processed by the YipAB phosphatases encoded directly downstream [76]. A careful genetic analysis revealed that the N-terminal portions of YitABC can be secreted through *Y. pestis*’s plasmid-encoded T3 injectisome, and that the N-terminal portion of YitB can be translocated into insect and mammalian cells in a T3SS-dependent manner [76]. These results suggest the surprising possibility that the Tc toxins of this organism are T3SS effectors. It has been noted that this mechanism for Tc export cannot be generalized to all entomopathogens, as *Xenorhabdus* spp., for instance, produce Tc toxins but have no T3SS [84].

### 4.8. Flagellar T3SS

In a few cases, the flagellar T3SS has been proposed to secrete non-flagellar proteins, usually following the observation that a secreted protein is absent from the culture supernatants of flagellar T3SS mutants [85,86,87]. This explanation should be invoked with caution, as flagellar assembly involves hierarchical gene expression and sequential activity of transcription factors that also control expression of non-flagellar genes, [88,89,90], potentially including those for dedicated secretion systems or the secreted proteins themselves. It is also worth clarifying that proteins secreted through a flagellar T3SS would be delivered to the extracellular space or attached to the bacterial surface, not directly injected into the host cell, as the flagellar T3SS lacks the translocation pore.

*Xenorhabdus* genomes lack obvious candidates for T3SS injectisomes [61], but the flagellar apparatus has been invoked in the secretion of several non-flagellar virulence factors in this organism. An *X. nematophila* mutant lacking FlhA, a structural component of the flagellar assembly apparatus, is defective in lipase activity, suggesting that the lipase, XlpA, is secreted through this structure [88]. The antibacterial xenocin complex XciA/XimB is also proposed to be secreted in this way [50], corroborated by the finding that the xenocin protein accumulates in FlhA mutant cells. Extensive mutational and transcriptional analysis has revealed a complex hierarchy of regulation linking motility and virulence gene expression (including expression of *xlpA*) in *Xenorhabdus* spp. [88,90,91], potentially complicating interpretation of these results.

### 4.9. Type 6 Secretion System: From the Bacterial Cytoplasm into a Target Cell

Like injectisome T3SSs, T6SSs deliver effector proteins directly into target cells; unlike T3SSs, the targets may be eukaryotic or bacterial [11,59]. Most of the characterized T6SSs are associated with antibacterial competition, some are involved in virulence, and a few contribute to symbiosis with host cells [60]. There is no clear secretion signal for T6-secreted proteins, but some share domains thought to interact with structural components at the tip of the T6SS, and others are fusions with the structural proteins VgrG or Hcp. 

Information on entomopathogen T6SSs comes mostly from bioinformatic predictions, and few specific effectors have been verified. *Photorhabdus* has four predicted T6SS gene clusters, which are located in pathogenicity islands [44]. *Xenorhabdus* genomes generally encode at least one T6SS [61], with some strains carrying a second, or even third [77]. Little is known about the functions of these secretion systems, though *X. bovienii* strains encoding multiple T6SSs tend to have a competitive advantage over strains that only encode one, suggesting a role in interbacterial competition [92]. An *X. nematophila* mutant lacking one of its two T6SSs retains virulence in insects [7], consistent with an antibacterial function. *Ps. entomophila* has two predicted T6SSs, which are proposed to play a role in interaction with insect hosts [93], but this has not yet been demonstrated. In *Y. pestis*, expression of T6SS structural proteins is increased when the bacteria are grown at 26 °C versus 37 °C, suggesting a role for T6 secretion in this organism’s association with its flea vector [94].

## 5. Other Secreted Proteins from Entomopathogens

In addition to these canonical secretion systems, entomopathogenic bacteria make use of some additional mechanisms for protein export, which we briefly describe here.

### 5.1. Modified Phage Tails: PVCs and the Antifeeding Prophage

Insecticidal surface structures related to phage are found in some entomopathogenic bacteria. For example, the numerous *Photorhabdus* virulence cassettes (PVCs) are genomic loci of 15–20 genes, including structural components related to phage tails and various “effectors” with similarity to diverse toxins, including Mcf and YopT. These assemble into needle-like structures on the bacterial surface and confer insecticidal activity on *E. coli* when expressed heterologously [95]. The *S. entomophila* antifeeding prophage (Afp) comprises similar structural elements and is responsible for the anti-feeding effect of *S. entomophila* on grass grubs (which is distinct from the symptoms of amber disease mediated by the SepABC toxin complex) [96,97]. PVCs, the AFP, and similar structures appear to be evolutionary intermediates between contractile phages and T6SSs [12,98]. For this reason, it is thought that they may inject effector toxins directly into host cells. Loci similar to PVCs and the Afp have since been identified in numerous bacteria with diverse lifestyles [98]. 

### 5.2. Tc Toxin Complexes

*Photorhabdus* spp. provided the first examples of a distinctive mechanism for transporting large toxins—the Tc toxin complexes—into target cells [29]. Tcs comprise three proteins: TcA, TcB, and TcC, which is the enzymatically active part of the Tc toxin [99]. Tcs have since been identified in a wide range of bacteria, many having no known association with insects [29]. In some entomopathogens, the Tc complex appears to remain attached to the bacterial cell surface, while in others it is released into the extracellular environment [61,62]. Little is yet known about how the large, signal-less Tc proteins are secreted from the bacterial cell. Speculations have been commendably reserved, but include nods to both T3SSs [76] and T6SSs [62], and it is possible that Tc export mechanism differ among different organisms. Recent structural analysis suggests that translocation into the host cell is achieved by the Tc complex itself. Cryo-EM studies reveal TcA oligomerizing into an unusual α-helix based channel in the host-cell membrane, likely facilitating entry of the TcC enzyme [99,100].

However they are secreted, Tc proteins frequently display striking oral and injectable toxicity towards insects [32,37,101,102,103] and are encoded in the genomes of many entomopathogens [104]. *S. entomophila*, the causative agent of amber disease in grass grubs, produces a Tc complex known as SepABC. Ingestion of the three Sep Tc proteins is sufficient to induce amber disease symptoms in *Costelytra zealandica* grass grubs [103]. *Y. entomophaga* mutants lacking this organism’s Tc are avirulent, and the purified toxin complex is orally toxic to a range of Coleopteran and Lepidopteran larvae [37]. *P. luminescens* and *P. asymbiotica* encode multiple Tc complexes, while *Ps. entomophila* and *X. nematophila* each encode at least one [5,84]. Strikingly, the *X. nematophila* Tc operon includes two distinct TcA genes, and these appear to specify toxicity towards particular insect species [102], supporting the notion that a given entomopathogen’s host range is influenced by its suite of Tcs [84].

## 6. Concluding Remarks

Entomopathogens use a wide range of mechanisms to secrete proteins involved in virulence, nutrient acquisition, and competition with other bacteria. Work on these proteins has shed light on new protein families relevant to many other organisms, as in the case of Tc toxins. Comparing secreted proteins from entomopathogens also illustrates pathogen evolution; secreted proteins that are obvious homologs may play different roles in pathogenesis, such as the hemolysins PhlA and XhlA, highlighting the adaptation of pathogens to different niches. Genomic analyses have provided many tantalizing candidates for novel virulence factors in entomopathogens. Defining their functions and mechanisms of secretion will enhance our understanding of insect pathogenic bacteria and expand our repertoire of potential biopesticides [18,29].

Entomopathogenic bacteria, in particular *Photorhabdus* spp. and *Xenorhabdus* spp., also release numerous non-protein molecules with interesting and potentially useful bioactivities into the extracellular environment [105,106,107,108]. How these secondary metabolites are synthesized is an area of active inquiry, but little is known about how they are exported from the bacterial cell. Filling in these details could facilitate industrial production of these molecules, some of which hold promise as pesticides or pharmaceuticals. Further studies on the secreted proteins and secondary metabolites of entomopathogenic bacteria are needed to better understand their biology and inform efforts to develop novel biopesticides.

## Figures and Tables

**Table 1:** Properties of bacterial secretion systems and distribution in entomopathogens.

			Features of Secreted Proteins			
Secretion System	Presence in Genome ^a^	Location	Destination	Signal Sequence	Folded/Unfolded	References
Sec	PL, PA, XN, XB, PsE ^b^, YE ^b^	IM	Periplasm	N-terminal, conserved	Unfolded	[3,10]
Tat	PL, PA, XN, XB, PsE ^b^, YE ^b^	IM	Periplasm	N-terminal, conserved	Folded	[3,10]
T1SS	PL, PA, XN, XB, PsE	IM, OM	Extracellular ^d^	C-terminal, conserved	Unfolded	[47,53,54]
T2SS	PL, XB, YE, PsE	IM, OM	Extracellular ^d^	Variable, may only appear in folded protein ^e^	Folded	[55]
T3SS	PL, PA, XN ^c^, XB ^c^, PsE ^c^, YE	IM, OM	Eukaryotic target cell	N-terminal, variable	Unfolded	[13,14,56]
T5aSS/autotransporter	PL, XN, XB, PsE, YE	OM	Extracellular ^d^	Structural features of passenger domain ^f^	Unfolded	[57,58]
T5bSS/TPS	PL, PA, XN, XB, PsE	OM	Extracellular ^d^	N-terminal, conserved ^f^	Unfolded	[57,58]
T6SS	PL, PA, XN, XB, PsE, YE	IM, OM	Bacterial or eukaryotic target cell	Unknown, may be fused to T6SS structural protein	Folded	[11,59,60]

PL: *Photorhabdus luminescens* TT01, PA: *Photorhabdus asymbiotica*, XN: *Xenorhabdus nematophila*, XB: *Xenorhabdus bovienii*, PsE: *Pseudomonas entomophila*, YE: *Yersinia entomophaga*. ^a^ Presence of secretion system in entomopathogen genome as reported in [4,6,7,8,43,61]. ^b^ The presence of Sec and Tat systems in *Pseudomonas entomophila* and *Yersinia entomophaga* genomes was verified by searching the GenBank records of their genomes (NZ_CP010029.1 and CT573326.1) for structural Sec and Tat elements. ^c^ Flagellar T3SS only. ^d^ Extracellular proteins may remain attached to the bacterial surface or be released into the environment. ^e^ Also have Sec or Tat signals to target to the periplasm. ^f^ Also have Sec signals to target to the periplasm.

**Table 2:** Examples of proteins secreted by entomopathogenic bacteria.

Bacterium	Secreted Protein	Activity	GenBank Identifier	Secretion System	Proposed Role in Pathogenesis	References
PA	Bla	β-lactamase	OCQ54324.1	Tat	β-lactam resistance	[52]
PL	Pdl/ORF54	Lipase	AAL18491.1	T2SS	Processing Tc toxins	[62]
XN	EstA	Lecithinase	CBJ90164.1	Autotransporter	Nutrient acquisition	[63]
PL	PhlA	Hemolysin	AL662784.1	TPS	Nutrient acquisition	[64]
XN	XhlA	Hemolysin	AAV33651.1	TPS	Immune evasion, contributes to virulence ^a^	[65]
XD	CdiA	Contact-dependent inhibition	CDG16822.1	TPS	Interbacterial competition	[66]
PL	Mcf1	Pro-apoptotic toxin	AAM88787.1	T1SS	Immune evasion, contributes to virulence ^b^	[67,68]
PL	PrtA	Metalloprotease	AAO39136.1	T1SS	Nutrient acquisition, immune evasion	[69,70]
XN	PrtA	Metalloprotease	CCW31579.1	T1SS	Immune evasion	[71]
PsE	AprA	Metalloprotease	CAK14412.1	T1SS	Immune evasion, contributes to virulence ^a^	[72,73]
PL	LopT	Cytotoxin	AAO18078.1	T3SS	Unknown, possible role in immune evasion	[74]
PL	Cif	Cycle inhibiting factor	CAE14889.1	T3SS	Unknown, likely host cell manipulation	[75]
YP	YitB	Cytotoxin	AAM83778.1	T3SS	Unknown, possible role in transmission	[76]

PA: *Photorhabdus asymbiotica*, PL: *Photorhabdus luminescens*, XN: *Xenorhabdus nematophila*, XD: *Xenorhabdus doucetiae*, PsE: *Pseudomonas entomophila*, YP: *Yersinia pestis*. ^a^ Deletion mutant defective in insect killing. ^b^ Heterologous expression confers insecticidal activity on *E. coli.*

## References

[1] Tseng T.-T., Tyler B.M., Setubal J.C. (2009). Protein secretion systems in bacterial-host associations, and their description in the Gene Ontology. BMC Microbiol..

[2] Costa T.R.D., Felisberto-Rodrigues C., Meir A., Prevost M.S., Redzej A., Trokter M., Waksman G. (2015). Secretion systems in Gram-negative bacteria: Structural and mechanistic insights. Nat. Rev. Microbiol..

[3] Gerlach R.G., Hensel M. (2007). Protein secretion systems and adhesins: The molecular armory of Gram-negative pathogens. Int. J. Med. Microbiol..

[4] Duchaud E., Rusniok C., Frangeul L., Buchrieser C., Givaudan A., Taourit S., Bocs S., Boursaux-Eude C., Chandler M., Charles J.-F. (2003). The genome sequence of the entomopathogenic bacterium *Photorhabdus luminescens*. Nat. Biotechnol..

[5] Vodovar N., Vallenet D., Cruveiller S., Rouy Z., Barbe V., Acosta C., Cattolico L., Jubin C., Lajus A., Segurens B. (2006). Complete genome sequence of the entomopathogenic and metabolically versatile soil bacterium *Pseudomonas entomophila*. Nat. Biotechnol..

[6] Wilkinson P., Waterfield N.R., Crossman L., Corton C., Sanchez-Contreras M., Vlisidou I., Barron A., Bignell A., Clark L., Ormond D. (2009). Comparative genomics of the emerging human pathogen *Photorhabdus asymbiotica* with the insect pathogen *Photorhabdus luminescens*. BMC Genom..

[7] Chaston J.M., Suen G., Tucker S.L., Andersen A.W., Bhasin A., Bode E., Bode H.B., Brachmann A.O., Cowles C.E., Cowles K.N. (2011). The *entomopathogenic* bacterial endosymbionts *Xenorhabdus* and *Photorhabdus*: Convergent lifestyles from divergent genomes. PLoS ONE.

[8] Hurst M.R.H., Beattie A., Altermann E., Moraga R.M., Harper L.A., Calder J., Laugraud A. (2016). The Draft Genome Sequence of the Yersinia *entomophaga Entomopathogenic* Type Strain MH96T. Toxins.

[9] Salmond G.P., Reeves P.J. (1993). Membrane traffic wardens and protein secretion in gram-negative bacteria. Trends Biochem. Sci..

[10] Green E.R., Mecsas J. (2016). Bacterial Secretion Systems: An Overview. Microbiol. Spectr..

[11] Filloux A. (2013). The rise of the Type VI secretion system. F1000prime Rep..

[12] Buttner C.R., Wu Y., Maxwell K.L., Davidson A.R. (2016). Baseplate assembly of phage Mu: Defining the conserved core components of contractile-tailed phages and related bacterial systems. Proc. Natl. Acad. Sci. USA.

[13] Diepold A., Armitage J.P. (2015). Type III secretion systems: The bacterial flagellum and the injectisome. Philos. Trans. R. Soc. Lond. B Biol. Sci..

[14] Deng W., Marshall N.C., Rowland J.L., McCoy J.M., Worrall L.J., Santos A.S., Strynadka N.C.J., Finlay B.B. (2017). Assembly, structure, function and regulation of type III secretion systems. Nat. Rev. Microbiol..

[15] Waterfield N.R., Daborn P.J., ffrench-Constant R.H. (2002). Genomic islands in *Photorhabdus*. Trends Microbiol..

[16] Vallet-Gely I., Lemaitre B., Boccard F. (2008). Bacterial strategies to overcome insect defences. Nat. Rev. Microbiol..

[17] Ruiu L. (2015). Insect Pathogenic Bacteria in Integrated Pest Management. Insects.

[18] Lacey L.A., Grzywacz D., Shapiro-Ilan D.I., Frutos R., Brownbridge M., Goettel M.S. (2015). Insect pathogens as biological control agents: Back to the future. J. Invertebr. Pathol..

[19] Adams B.J., Fodor A., Koppenhöfer H.S., Stackebrandt E., Stock S.P., Klein M.G. (2006). Biodiversity and systematics of nematode-bacterium *entomopathogens*. Biol. Control.

[20] Forst S., Dowds B., Boemare N., Stackebrandt E. (1997). *Xenorhabdus* and *Photorhabdus* spp.: Bugs that kill bugs. Annu. Rev. Microbiol..

[21] Boemare N.E., Gaugler R. (2002). Biology, taxonomy and systematics of *Photorhabdus* and *Xenorhabdus*. Entomopathogenic Nematology.

[22] Stock S.P., Goodrich-Blair H. (2008). *Entomopathogenic nematodes* and their bacterial symbionts: The inside out of a mutualistic association. Symbiosis.

[23] Kaya H.K., Gaugler R. (1993). *Entomopathogenic* *nematodes*. Annu. Rev. Entomol..

[24] Grewal P.S., Grewal S.K., Malik V.S., Klein M.G. (2010). Differences in susceptibility of introduced and native white grub species to *entomopathogenic* nematodes from various geographic localities. Biol. Control.

[25] Chen S., Li J., Han X., Moens M. (2003). Effect of temperature on the pathogenicity of *entomopathogenic* nematodes (*Steinernema* and *Heterorhabditis* spp.) to Delia radicum. BioControl.

[26] Toepfer S., Gueldenzoph C., Ehlers R.-U., Kuhlmann U. (2005). Screening of *entomopathogenic* nematodes for virulence against the invasive western corn rootworm, Diabrotica virgifera virgifera (*Coleoptera*: *Chrysomelidae*) in Europe. Bull. Entomol. Res..

[27] Koppenhöfer A.M., Grewal P.S., Fuzy E.M. (2006). Virulence of the *entomopathogenic* nematodes *Heterorhabditis bacteriophora*, *Heterorhabditis zealandica*, and *Steinernema* scarabaei against five white grub species (*Coleoptera*: *Scarabaeidae*) of economic importance in turfgrass in North America. Biol. Control.

[28] Shapiro-Ilan D.I., Han R., Dolinksi C. (2012). *Entomopathogenic nematode* production and application technology. J. Nematol..

[29] ffrench-Constant R.H., Dowling A., Waterfield N.R. (2007). Insecticidal toxins from *Photorhabdus* bacteria and their potential use in agriculture. Toxicon.

[30] ffrench-Constant R.H., Bowen D.J. (2000). Novel insecticidal toxins from nematode-symbiotic bacteria. Cell. Mol. Life Sci..

[31] Brown S.E., Cao A.T., Hines E.R., Akhurst R.J., East P.D. (2004). A novel secreted protein toxin from the insect pathogenic bacterium *Xenorhabdus* nematophila. J. Biol. Chem..

[32] Waterfield N., Hares M., Yang G., Dowling A., ffrench-Constant R. (2005). Potentiation and cellular phenotypes of the insecticidal Toxin complexes of *Photorhabdus* bacteria. Cell. Microbiol..

[33] Gerrard J., Waterfield N., Vohra R., ffrench-Constant R. (2004). Human infection with *Photorhabdus asymbiotica*: An emerging bacterial pathogen. Microbes Infect..

[34] Brugirard-Ricaud K., Givaudan A., Parkhill J., Boemare N., Kunst F., Zumbihl R., Duchaud E. (2004). Variation in the effectors of the type III secretion system among *Photorhabdus* species as revealed by genomic analysis. J. Bacteriol..

[35] Tounsi S., Blight M., Jaoua S., de Lima Pimenta A. (2006). From insects to human hosts: Identification of major genomic differences between *entomopathogenic* strains of *Photorhabdus* and the emerging human pathogen *Photorhabdus asymbiotica*. Int. J. Med. Microbiol..

[36] Jackson T.A., Boucias D.G., Thaler J.O. (2001). Pathobiology of amber disease, caused by *Serratia* Spp., in the New Zealand grass grub, *Costelytra zealandica*. J. Invertebr. Pathol..

[37] Hurst M.R.H., Jones S.A., Binglin T., Harper L.A., Jackson T.A., Glare T.R. (2011). The main virulence determinant of Yersinia *entomophaga* MH96 is a broad-host-range toxin complex active against insects. J. Bacteriol..

[38] Hinnebusch B.J., Rudolph A.E., Cherepanov P., Dixon J.E., Schwan T.G., Forsberg A. (2002). Role of Yersinia murine toxin in survival of *Yersinia pestis* in the midgut of the flea vector. Science.

[39] Hinchliffe S.J., Isherwood K.E., Stabler R.A., Prentice M.B., Rakin A., Nichols R.A., Oyston P.C.F., Hinds J., Titball R.W., Wren B.W. (2003). Application of DNA microarrays to study the evolutionary genomics of *Yersinia pestis* and *Yersinia pseudotuberculosis*. Genome Res..

[40] Vodovar N., Vinals M., Liehl P., Basset A., Degrouard J., Spellman P., Boccard F., Lemaitre B. (2005). *Drosophila* host defense after oral infection by an *entomopathogenic Pseudomonas* species. Proc. Natl. Acad. Sci. USA.

[41] Martinez-Garcia P.M., Ramos C., Rodriguez-Palenzuela P. (2015). T346Hunter: A novel web-based tool for the prediction of type III, type IV and type VI secretion systems in bacterial genomes. PLoS ONE.

[42] Abby S.S., Rocha E.P.C. (2017). Identification of Protein Secretion Systems in Bacterial Genomes Using MacSyFinder. Methods Mol. Biol..

[43] Abby S.S., Cury J., Guglielmini J., Neron B., Touchon M., Rocha E.P.C. (2016). Identification of protein secretion systems in bacterial genomes. Sci. Rep..

[44] Rodou A., Ankrah D.O., Stathopoulos C. (2010). Toxins and secretion systems of *Photorhabdus luminescens*. Toxins.

[45] Bendtsen J.D., Nielsen H., Widdick D., Palmer T., Brunak S. (2005). Prediction of twin-arginine signal peptides. BMC Bioinform..

[46] Nielsen H. (2017). Predicting Secretory Proteins with SignalP. Methods Mol. Biol..

[47] Delepelaire P. (2004). Type I secretion in gram-negative bacteria. Biochim. Biophys. Acta.

[48] An Y., Wang J., Li C., Leier A., Marquez-Lago T., Wilksch J., Zhang Y., Webb G.I., Song J., Lithgow T. (2018). Comprehensive assessment and performance improvement of effector protein predictors for bacterial secretion systems III, IV and VI. Brief. Bioinform..

[49] Maffei B., Francetic O., Subtil A. (2017). Tracking proteins secreted by bacteria: What’s in the toolbox?. Front. Cell. Infect. Microbiol..

[50] Singh P., Park D., Forst S., Banerjee N. (2013). Xenocin export by the flagellar type III pathway in *Xenorhabdus* nematophila. J. Bacteriol..

[51] Gerrard J.G., Stevens R.P. (2017). A review of clinical cases of infection with *Photorhabdus* asymbiotica. Curr. Top. Microbiol. Immunol..

[52] Schriefer E.-M., Hoffmann-Thoms S., Schmid F.X., Schmid A., Heesemann J. (2013). *Yersinia enterocolitica* and *Photorhabdus asymbiotica* beta-lactamases BlaA are exported by the twin-arginine translocation pathway. Int. J. Med. Microbiol..

[53] Kanonenberg K., Schwarz C.K.W., Schmitt L. (2013). Type I secretion systems—A story of appendices. Res. Microbiol..

[54] Holland I.B., Peherstorfer S., Kanonenberg K., Lenders M., Reimann S., Schmitt L. (2016). Type I protein secretion-deceptively simple yet with a wide range of mechanistic variability across the family. EcoSal Plus.

[55] Korotkov K.V., Sandkvist M., Hol W.G.J. (2012). The type II secretion system: Biogenesis, molecular architecture and mechanism. Nat. Rev. Microbiol..

[56] Troisfontaines P., Cornelis G.R. (2005). Type III secretion: More systems than you think. Physiology.

[57] Leo J.C., Grin I., Linke D. (2012). Type V secretion: Mechanism(s) of autotransport through the bacterial outer membrane. Philos. Trans. R. Soc. Lond. B Biol. Sci..

[58] Leyton D.L., Rossiter A.E., Henderson I.R. (2012). From self sufficiency to dependence: Mechanisms and factors important for autotransporter biogenesis. Nat. Rev. Microbiol..

[59] Bingle L.E., Bailey C.M., Pallen M.J. (2008). Type VI secretion: A beginner’s guide. Curr. Opin. Microbiol..

[60] Russell A.B., Peterson S.B., Mougous J.D. (2014). Type VI secretion system effectors: Poisons with a purpose. Nat. Rev. Microbiol..

[61] Silva C.P., Waterfield N.R., Daborn P.J., Dean P., Chilver T., Au C.P.Y., Sharma S., Potter U., Reynolds S.E., ffrench-Constant R.H. (2002). Bacterial infection of a model insect: *Photorhabdus luminescens* and *Manduca sexta*. Cell. Microbiol..

[62] Yang G., Hernandez-Rodriguez C.S., Beeton M.L., Wilkinson P., Ffrench-Constant R.H., Waterfield N.R. (2012). Pdl1 is a putative lipase that enhances *Photorhabdus* toxin complex secretion. PLoS Pathog..

[63] Richards G.R., Goodrich-Blair H. (2010). Examination of *Xenorhabdus* nematophila lipases in pathogenic and mutualistic host interactions reveals a role for xlpA in nematode progeny production. Appl. Environ. Microbiol..

[64] Brillard J., Duchaud E., Boemare N., Kunst F., Givaudan A. (2002). The PhlA hemolysin from the *entomopathogenic* bacterium *Photorhabdus luminescens* belongs to the two-partner secretion family of hemolysins. J. Bacteriol..

[65] Cowles K.N., Goodrich-Blair H. (2005). Expression and activity of a *Xenorhabdus* nematophila haemolysin required for full virulence towards *Manduca sexta* insects. Cell. Microbiol..

[66] Ogier J.-C., Duvic B., Lanois A., Givaudan A., Gaudriault S. (2016). A new member of the growing family of contact-dependent growth inhibition systems in *Xenorhabdus* doucetiae. PLoS ONE.

[67] Daborn P.J., Waterfield N., Silva C.P., Au C.P.Y., Sharma S., Ffrench-Constant R.H. (2002). A single *Photorhabdus* gene, makes caterpillars floppy (MCF), allows *Escherichia coli* to persist within and kill insects. Proc. Natl. Acad. Sci. USA.

[68] Dowling A.J., Daborn P.J., Waterfield N.R., Wang P., Streuli C.H., ffrench-Constant R.H. (2004). The insecticidal toxin Makes caterpillars floppy (MCF) promotes apoptosis in mammalian cells. Cell. Microbiol..

[69] Bowen D.J., Rocheleau T.A., Grutzmacher C.K., Meslet L., Valens M., Marble D., Dowling A., Ffrench-Constant R., Blight M.A. (2003). Genetic and biochemical characterization of PrtA, an RTX-like metalloprotease from *Photorhabdus*. Microbiology.

[70] Felfoldi G., Marokhazi J., Kepiro M., Venekei I. (2009). Identification of natural target proteins indicates functions of a serralysin-type metalloprotease, PrtA, in anti-immune mechanisms. Appl. Environ. Microbiol..

[71] Caldas C., Cherqui A., Pereira A., Simoes N. (2002). Purification and characterization of an extracellular protease from *Xenorhabdus* nematophila involved in insect immunosuppression. Appl. Environ. Microbiol..

[72] Liehl P., Blight M., Vodovar N., Boccard F., Lemaitre B. (2006). Prevalence of local immune response against oral infection in a *Drosophila*/*Pseudomonas* infection model. PLoS Pathog..

[73] Lee S.A., Jang S.H., Kim B.H., Shibata T., Yoo J., Jung Y., Kawabata S.-I., Lee B.L. (2018). Insecticidal activity of the metalloprotease AprA occurs through suppression of host cellular and humoral immunity. Dev. Comp. Immunol..

[74] Brugirard-Ricaud K., Duchaud E., Givaudan A., Girard P.A., Kunst F., Boemare N., Brehelin M., Zumbihl R. (2005). Site-specific antiphagocytic function of the *Photorhabdus luminescens* type III secretion system during insect colonization. Cell. Microbiol..

[75] Jubelin G., Chavez C.V., Taieb F., Banfield M.J., Samba-Louaka A., Nobe R., Nougayrede J.-P., Zumbihl R., Givaudan A., Escoubas J.-M. (2009). Cycle inhibiting factors (CIFs) are a growing family of functional cyclomodulins present in invertebrate and mammal bacterial pathogens. PLoS ONE.

[76] Gendlina I., Held K.G., Bartra S.S., Gallis B.M., Doneanu C.E., Goodlett D.R., Plano G.V., Collins C.M. (2007). Identification and type III-dependent secretion of the *Yersinia pestis* insecticidal-like proteins. Mol. Microbiol..

[77] Kim I.-H., Aryal S.K., Aghai D.T., Casanova-Torres A.M., Hillman K., Kozuch M.P., Mans E.J., Mauer T.J., Ogier J.-C., Ensign J.C. (2017). The insect pathogenic bacterium *Xenorhabdus innexi* has attenuated virulence in multiple insect model hosts yet encodes a potent mosquitocidal toxin. BMC Genom..

[78] Ruhe Z.C., Low D.A., Hayes C.S. (2013). Bacterial contact-dependent growth inhibition. Trends Microbiol..

[79] Thomas S., Holland I.B., Schmitt L. (2014). The Type 1 secretion pathway—The hemolysin system and beyond. Biochim. Biophys. Acta.

[80] Linhartova I., Bumba L., Masin J., Basler M., Osicka R., Kamanova J., Prochazkova K., Adkins I., Hejnova-Holubova J., Sadilkova L. (2010). RTX proteins: A highly diverse family secreted by a common mechanism. FEMS Microbiol. Rev..

[81] Mulley G., Beeton M.L., Wilkinson P., Vlisidou I., Ockendon-Powell N., Hapeshi A., Tobias N.J., Nollmann F.I., Bode H.B., van den Elsen J. (2015). From Insect to Man: *Photorhabdus* Sheds Light on the Emergence of Human Pathogenicity. PLoS ONE.

[82] Ogier J.-C., Pages S., Bisch G., Chiapello H., Medigue C., Rouy Z., Teyssier C., Vincent S., Tailliez P., Givaudan A. (2014). Attenuated virulence and genomic reductive evolution in the *entomopathogenic* bacterial symbiont species, *Xenorhabdus poinarii*. Genome Biol. Evol..

[83] Murfin K.E., Whooley A.C., Klassen J.L., Goodrich-Blair H. (2015). Comparison of *Xenorhabdus* bovienii bacterial strain genomes reveals diversity in symbiotic functions. BMC Genom..

[84] Hinchliffe S.J., Hares M.C., Dowling A.J., ffrench-Constant R.H. (2010). Insecticidal Toxins from the *Photorhabdus* and *Xenorhabdus* Bacteria. Open Toxinol. J..

[85] Young G.M., Schmiel D.H., Miller V.L. (1999). A new pathway for the secretion of virulence factors by bacteria: The flagellar export apparatus functions as a protein-secretion system. Proc. Natl. Acad. Sci. USA.

[86] Konkel M.E., Klena J.D., Rivera-Amill V., Monteville M.R., Biswas D., Raphael B., Mickelson J. (2004). Secretion of virulence proteins from Campylobacter jejuni is dependent on a functional flagellar export apparatus. J. Bacteriol..

[87] Chaban B., Hughes H.V., Beeby M. (2015). The flagellum in bacterial pathogens: For motility and a whole lot more. Semin. Cell Dev. Biol..

[88] Park D., Forst S. (2006). Co-regulation of motility, exoenzyme and antibiotic production by the EnvZ-OmpR-FlhDC-FliA pathway in *Xenorhabdus* nematophila. Mol. Microbiol..

[89] Duan Q., Zhou M., Zhu L., Zhu G. (2013). Flagella and bacterial pathogenicity. J. Basic Microbiol..

[90] Givaudan A., Lanois A. (2017). Flagellar Regulation and Virulence in the *Entomopathogenic* Bacteria-*Xenorhabdus* nematophila and *Photorhabdus luminescens*. Curr. Top. Microbiol. Immunol..

[91] Richards G.R., Goodrich-Blair H. (2009). Masters of conquest and pillage: *Xenorhabdus* nematophila global regulators control transitions from virulence to nutrient acquisition. Cell. Microbiol..

[92] McMullen J.G., McQuade R., Ogier J.-C., Pages S., Gaudriault S., Patricia Stock S. (2017). Variable virulence phenotype of *Xenorhabdus* bovienii (*gamma-Proteobacteria*: *Enterobacteriaceae*) in the absence of their vector hosts. Microbiology.

[93] Sarris P.F., Scoulica E.V. (2011). *Pseudomonas entomophila* and *Pseudomonas mendocina*: Potential models for studying the bacterial type VI secretion system. Infect. Genet. Evol. J. Mol. Epidemiol. Evol. Genet. Infect. Dis..

[94] Pieper R., Huang S.-T., Robinson J.M., Clark D.J., Alami H., Parmar P.P., Perry R.D., Fleischmann R.D., Peterson S.N. (2009). Temperature and growth phase influence the outer-membrane proteome and the expression of a type VI secretion system in *Yersinia pestis*. Microbiology.

[95] Yang G., Dowling A.J., Gerike U., ffrench-Constant R.H., Waterfield N.R. (2006). *Photorhabdus* virulence cassettes confer injectable insecticidal activity against the wax moth. J. Bacteriol..

[96] Hurst M.R.H., Glare T.R., Jackson T.A. (2004). Cloning *Serratia entomophila* antifeeding genes—A putative defective prophage active against the grass grub *Costelytra zealandica*. J. Bacteriol..

[97] Heymann J.B., Bartho J.D., Rybakova D., Venugopal H.P., Winkler D.C., Sen A., Hurst M.R.H., Mitra A.K. (2013). Three-dimensional structure of the toxin-delivery particle antifeeding prophage of *Serratia entomophila*. J. Biol. Chem..

[98] Sarris P.F., Ladoukakis E.D., Panopoulos N.J., Scoulica E.V. (2014). A phage tail-derived element with wide distribution among both prokaryotic domains: A comparative genomic and phylogenetic study. Genome Biol. Evol..

[99] Gatsogiannis C., Lang A.E., Meusch D., Pfaumann V., Hofnagel O., Benz R., Aktories K., Raunser S. (2013). A syringe-like injection mechanism in *Photorhabdus luminescens* toxins. Nature.

[100] Gatsogiannis C., Merino F., Prumbaum D., Roderer D., Leidreiter F., Meusch D., Raunser S. (2016). Membrane insertion of a TC toxin in near-atomic detail. Nat. Struct. Mol. Biol..

[101] Bowen D., Blackburn M., Rocheleau T., Grutzmacher C., ffrench-Constant R.H. (2000). Secreted proteases from *Photorhabdus luminescens*: Separation of the extracellular proteases from the insecticidal TC toxin complexes. Insect Biochem. Mol. Biol..

[102] Sergeant M., Jarrett P., Ousley M., Morgan J.A.W. (2003). Interactions of insecticidal toxin gene products from *Xenorhabdus* nematophilus PMFI296. Appl. Environ. Microbiol..

[103] Hurst M.R.H., Jones S.M., Tan B., Jackson T.A. (2007). Induced expression of the *Serratia entomophila* Sep proteins shows activity towards the larvae of the New Zealand grass grub *Costelytra zealandica*. FEMS Microbiol. Lett..

[104] Waterfield N.R., Bowen D.J., Fetherston J.D., Perry R.D., ffrench-Constant R.H. (2001). The TC genes of *Photorhabdus*: A growing family. Trends Microbiol..

[105] Lang G., Kalvelage T., Peters A., Wiese J., Imhoff J.F. (2008). Linear and cyclic peptides from the *entomopathogenic* bacterium *Xenorhabdus* nematophilus. J. Nat. Prod..

[106] Bode H.B. (2009). *Entomopathogenic* bacteria as a source of secondary metabolites. Curr. Opin. Chem. Biol..

[107] Tobias N.J., Wolff H., Djahanschiri B., Grundmann F., Kronenwerth M., Shi Y.-M., Simonyi S., Grun P., Shapiro-Ilan D., Pidot S.J. (2017). Natural product diversity associated with the nematode symbionts *Photorhabdus* and *Xenorhabdus*. Nat. Microbiol..

[108] Stock S.P., Kusakabe A., Orozco R.A. (2017). Secondary metabolites produced by *Heterorhabditis* symbionts and their application in agriculture: What we know and what to do next. J. Nematol..

